# Supplementary material for: Clinical utility of plasma Aβ42/40 ratio by LC-MS/MS in Alzheimer’s disease assessment
Source: Front Neurol. 2024 Mar 25;15:1364658. doi: 10.3389/fneur.2024.1364658 (PMC11003272; doi:10.3389/fneur.2024.1364658)
Supplement: Supplementary file 1 [file Table_1.DOCX]

**Supplemental Online Content**

Weber DM, Taylor SW, Lagier RJ, et al. Clinical Utility of Plasma Aβ42/40 Ratio by LC-MS/MS in Alzheimer’s Disease Assessment

**Table S1.** ADRC participant characteristics by cognitive diagnosis and amyloid PET status

Aβ, beta-amyloid; CDR, clinical dementia rating; MMSE, mini-mental state examination; NA, not applicable; PET, positron emission tomography; SD, standard deviation; SUVR, standard uptake value ratio

^a^ ANOVA analysis performed on HC Aβ-/+, MCI Aβ-/+, and AD Aβ+ groups only

^b^ ANOVA analysis performed on Combined HC, MCI, and AD groups

^c^ For continuous variables global *P*-value from ANOVA F-test. Otherwise, Fisher's exact test

**Table S2:** Comparison of mass spectrometry-based methods for plasma Aβ42/40

| **Plasma Assay** | ***Quest IP-LC-MS/MS*** | **Washington University IP-LC-MS/MS** | **Araclon-LC-MS/MS** | **Shimadzu IP-MALDI TOF** | **UGOT IP-LC-MS/MS** |
| --- | --- | --- | --- | --- | --- |
| **Specimen volume (mL)** | *0.50* | 0.45 | 0.20 | 0.25 | 0.25 |
| **Sample preparation** | *IP, digestion with Lys-C* | IP, digestion with Lys-R | IP-free, no digestion | Sequential IP, no digestion | Dual IP, no digestion |
| **Aβ sequence detected** | *Aβ40: 29-40*  *Aβ42: 29-42* | Aβ40: 28-40  Aβ42: 28-42 | Aβ40: 1-40  Aβ42: 1-42 | Aβ40: 1-40  Aβ42: 1-42 APP: 669-711 | Aβ40: 1-40 Aβ42: 1-42 |
| **HPLC** | *Thermo Transcend Vanquish TLX-4 UPLC* | Waters Acquity M-Class UPLC | Sciex M3 MicroLC System | NA | Thermo Dionex UltiMate 3000 |
| **Multiplex** | *Yes; 4-plex UPLC* | No; single-plex UPLC | No; single-plex UPLC | NA | Partial; 2-plex UPLC |
| **Mass Spectrometer** | *Thermo Scientific Altis Plus* | Thermo Scientific Fusion Lumos Tribrid | Sciex QTRAP 6500+ with SelexION ion mobility system | Shimadzu AXIMA Performance | Thermo Scientific Q Exactive |
| **MS Type** | *Triple quadrupole, low resolution* | Orbitrap, high resolution | Triple quadrupole, low resolution | MALDI TOF/TOF, high resolution | Orbitrap, high resolution |
| **MS list price (USD)** | *$508,000* | $1,164,000 | $708,422 | $512,658 | $630,000 ^a^ |
| **Throughput** | *4 min/sample* | 6 min/sample | 9 min/sample | NR | 14.4 min/sample |
| **Automated workflow** | *Yes-fully automated sample preparation* | NR | NR | NR | NR |
| **Clinical studies** | *See Table S3* | Multiple | Multiple | Multiple | Multiple |
| **Aβ42/40 ratio fold change between groups** | *18%* | 7% - 14% | 11% - 18% | 8% - 14% | 11% - 21% |
| **Aβ42/40 ratio cutoff** | *0.160* | 0.089 - 0.125 | 0.241 | 0.0362 - 0.0392 ^c^ | 0.095 |
| **Prevalence of Aβ PET positivity** | *40%* | 25%-65% | 18%-50% | 41%-54% | 19%-50% |
| **ROC-AUC range** ^b^ | *0.84* | 0.79 - 0.89 | 0.74-0.87 | 0.72-0.97 | 0.63-0.82 |
| **Sensitivity** ^b^ | *91%* | 73% - 90% | 86% | 66% - 96% | 87% |
| **Specificity** ^b^ | *76%* | 63% - 88% | 81% | 87% - 92% | 72% |
| **Inter-assay precision: Aβ40** | *4% - 6%* | 3% - 8% | 4% - 5% | 9% - 11% | 3% - 7% |
| **Inter-assay precision: Aβ42** | *4% - 8%* | 3% - 10% | 5% - 8% | 6% - 17% | 7% - 15% |
| **Inter-assay precision: Aβ42/40 ratio (average)** | *<6%* | <4% | NR | 10% | NR |
| **Bias: Aβ40** | *11%* | NR | -7% to -6% | NR | NR |
| **Bias: Aβ42** | *12%* | NR | -6% to -3% | NR | NR |
| **Bias: Aβ42/40 ratio** | *<1%* | <1% | NR | NR | NR |
| **Linear range (pg/mL)** | *Aβ40: 10 – 2,500*  *Aβ42: 10 – 2,500* | Aβ40: 10 – 1,780  Aβ42: 2 - 254 | Aβ40: 50 – 1,000  Aβ42: 10 - 200 | Aβ40: 43 - 693  Aβ42: 11 - 180 | Aβ40: 20 - 400  Aβ42: 5 - 100 |
| **Calibrator Matrix** | *Human plasma-charcoal stripped* | HSA in PBS | Human plasma | BSA in PBS | BSA in PBS |
| **References** | *This work, [1]* | [2-11] | [8, 12, 13] | [8-10, 14] | [8-10, 15] |

Aβ, beta-amyloid; BSA, bovine serum albumin; HSA, human serum albumin; HPLC, high-performance liquid chromatography; IP, immunoprecipitation; MS, mass spectrometry; NA, not applicable; NR, not reported; PBS, phosphate-buffered saline; PET, positron emission tomography; UGOT, University of Gothenburg

^a^ Model discontinued; listed price is for a refurbished instrument

^b^ Ranges reported from multiple cohorts; comparisons of clinical performance measures are most valid when methods are compared using specimens from the same cohort (see Discussion) [16]

^c^ Study reports Aβ40/42; values converted to Aβ42/40

**Table S3:** Comparison of recent studies with ~40% of PET positivity

| **Assay type** | **Clinical studies** | **Prevalence of PET+, %** | **Cohort size** | **Predominant race/**  **ethnicity** | **HC/**  **MCI/AD/**  **nonAD** | **Age** | | | | | **AUC (95% CI)** | **Ref** |
| --- | --- | --- | --- | --- | --- | --- | --- | --- | --- | --- | --- | --- |
|  |  |  |  |  |  | **Aβ-, N** | | **Aβ+, N** | | ***P*-value** |  |  |
| *IP-LC-MS/MS* | *1Florida Alzheimer’s Disease Research Center (US)* | *40* | *250* | *Hispanic (60%)* | *72/124/54/0* | *72y, 149* | *73y, 101* | | *0.15* | | *0.84 (0.79-0.89)* | *this work* |
| Multiple comparisons | BioFinder (Sweden) | 38 | 286 | White | 182/104/0/0 | 71y^a^, 176 | 74y^a^, 110 | | 0.001 | |  | Janelidze et al 2021 [8] |
| IP-LC-MS/MS | |  |  |  |  |  |  | |  | | 0.83 (0.79-0.88) | |
| LC-MS/MS |  |  |  |  |  |  |  | |  | | 0.75 (0.70-0.81) | |
| ECL |  |  |  |  |  |  |  | |  | | 0.73 (0.67-0.78) | |
| ELISA |  |  |  |  |  |  |  | |  | | 0.67 (0.61-0.74) | |
| SIMOA |  |  |  |  |  |  |  | |  | | 0.66 (0.59-0.72) | |
| IP-LC-MS/MS | Multiple (US) | 39 | 414 | White (66%)/ non-Hispanic (77%) | NR | 68y, 253 | 74y, 161 | | <.001 | | 0.81 (0.77-0.85) | West et al 2021 [2] |
| CLEIA | Keio University (Japan) | 37 | 174 | Asian | 58/43/32/41 | 69y, 109 | 75y, 65 | | <.001 | | 0.95 (0.92-0.98) | Bun et al 2023 [17] |

Aβ-, amyloid beta negative; Aβ+, amyloid beta positive; CLEIA, chemiluminescence enzyme immunoassay; ECL, electrochemiluminescence immunoassay; ELISA, enzyme-linked immunosorbent assay; IP, immunoprecipitation; LC-MS/MS, liquid chromatography-tandem mass spectrometry; SIMOA, ultrasensitive single molecule array assays.

^a^ Median age. All other studies are reported as mean age.

**Figure S1.** Correlation and diagnostic performance of the Aβ42/40 ratio with cognitive diagnosis for the ADRC cohort. **A)** Plasma Aβ42/40 ratio compared with healthy controls (ADRC-HC), mild cognitive impairment (ADRC-MCI), and Alzheimer’s disease (ADRC-AD). Amyloid PET negative (Aβ-PET-) individuals are illustrated with blue circles, and amyloid PET positive (Aβ-PET+) individuals are illustrated with orange circles; **B)** ROC-AUC of the plasma Aβ42/40 ratio for ADRC-HC vs. ADRC-AD. *a* = Significant at *P* < 0.001


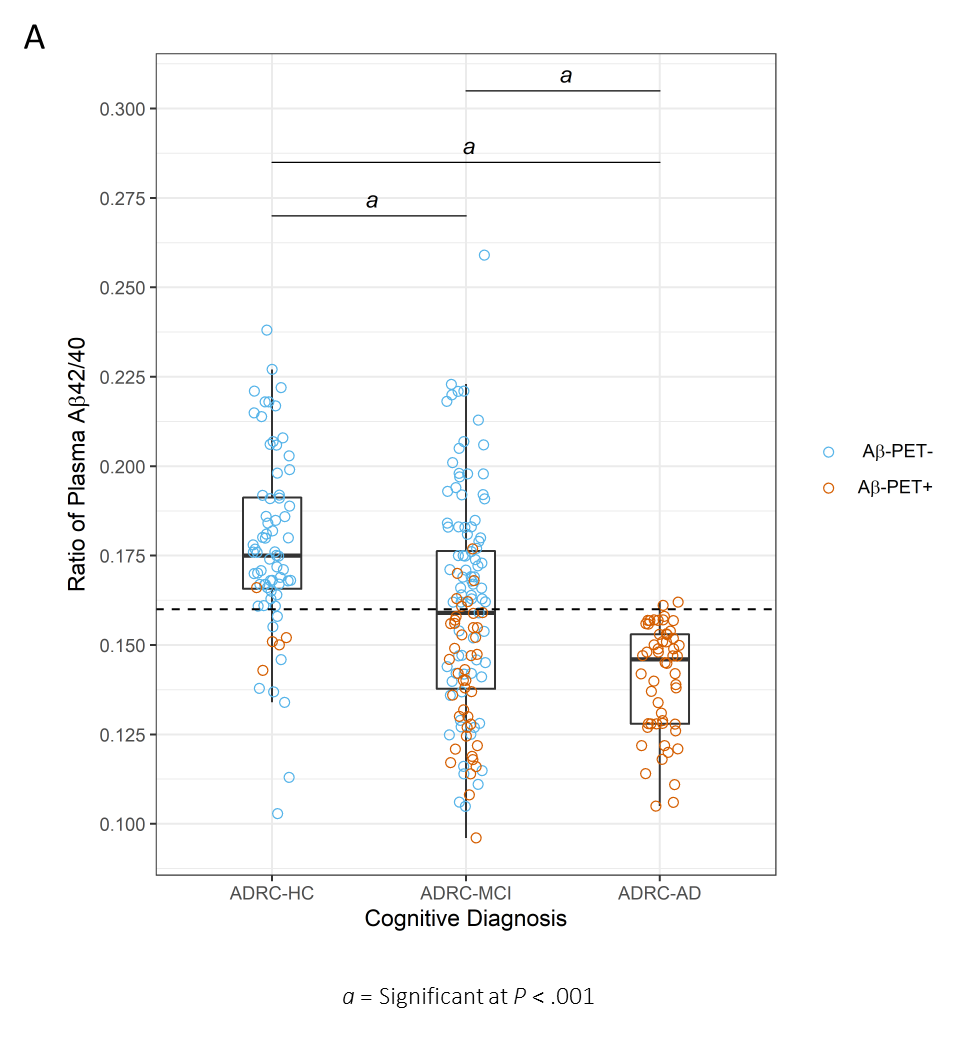


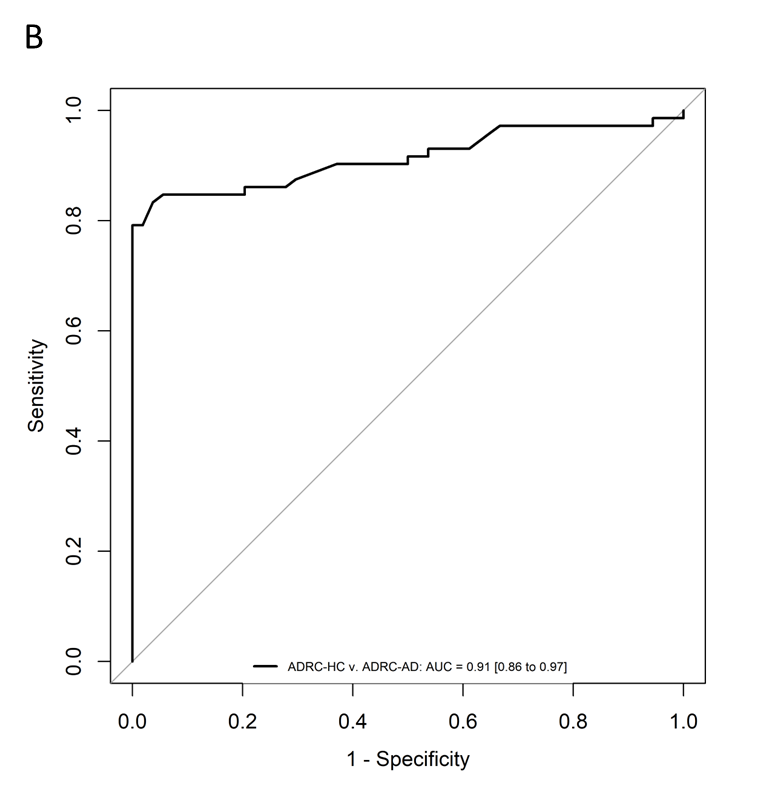


**Figure S2.** Plasma Aβ42/40 ratio in Alzheimer’s disease (ADRC-AD) participants categorized (A) and correlated (B) by MMSE scores and categorized (C) and correlated (D) by CDR scores indicative of severity of dementia. No linear correlation (r is Pearson correlation coefficient) between the Aβ42/40 ratio and MMSE (*P* = .17) or CDR (*P* = .26) was detected. No between group differences in mean Aβ42/40 ratio were detected for either MMSE classification (*P* = .58) or CDR classification (*P* = .69).


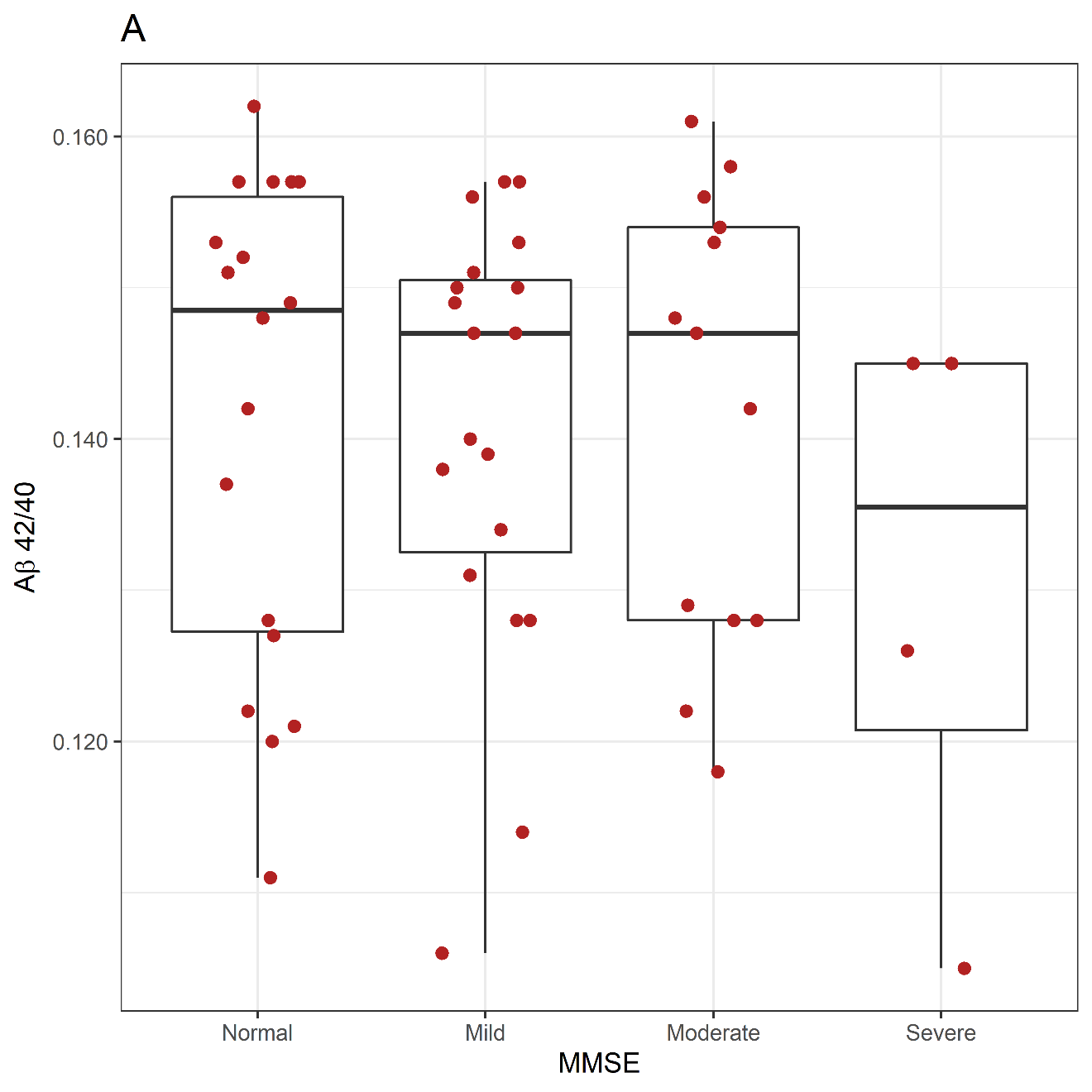


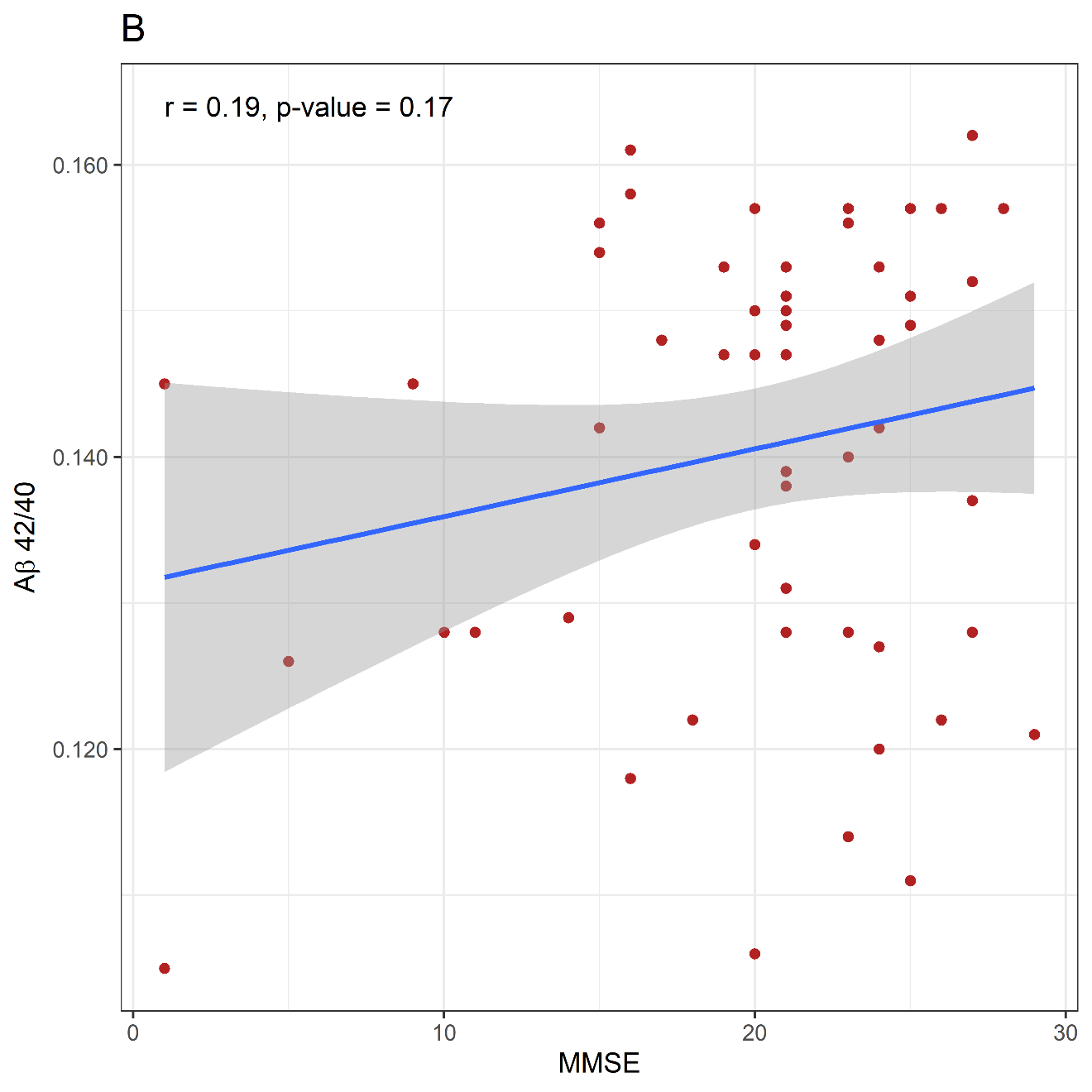


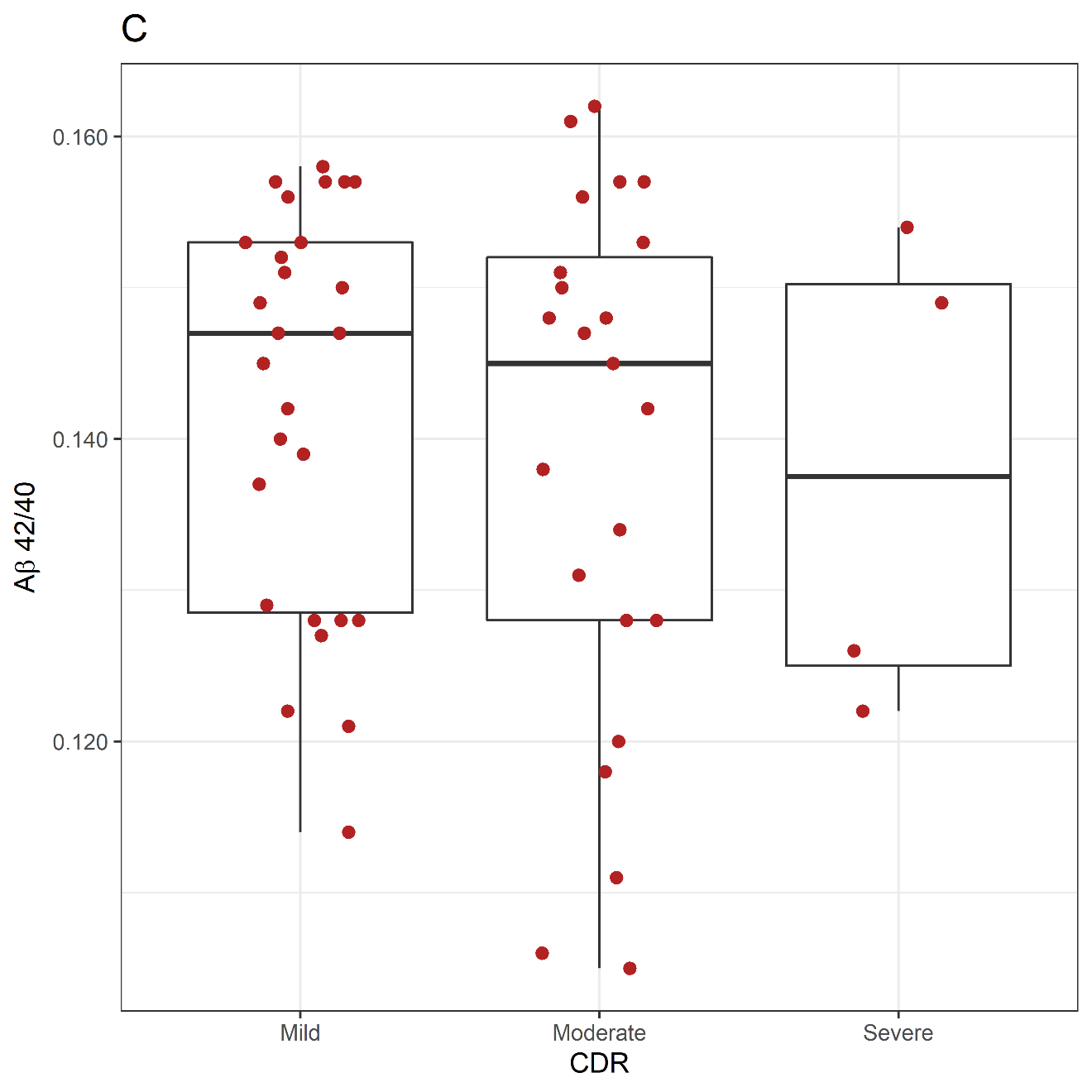


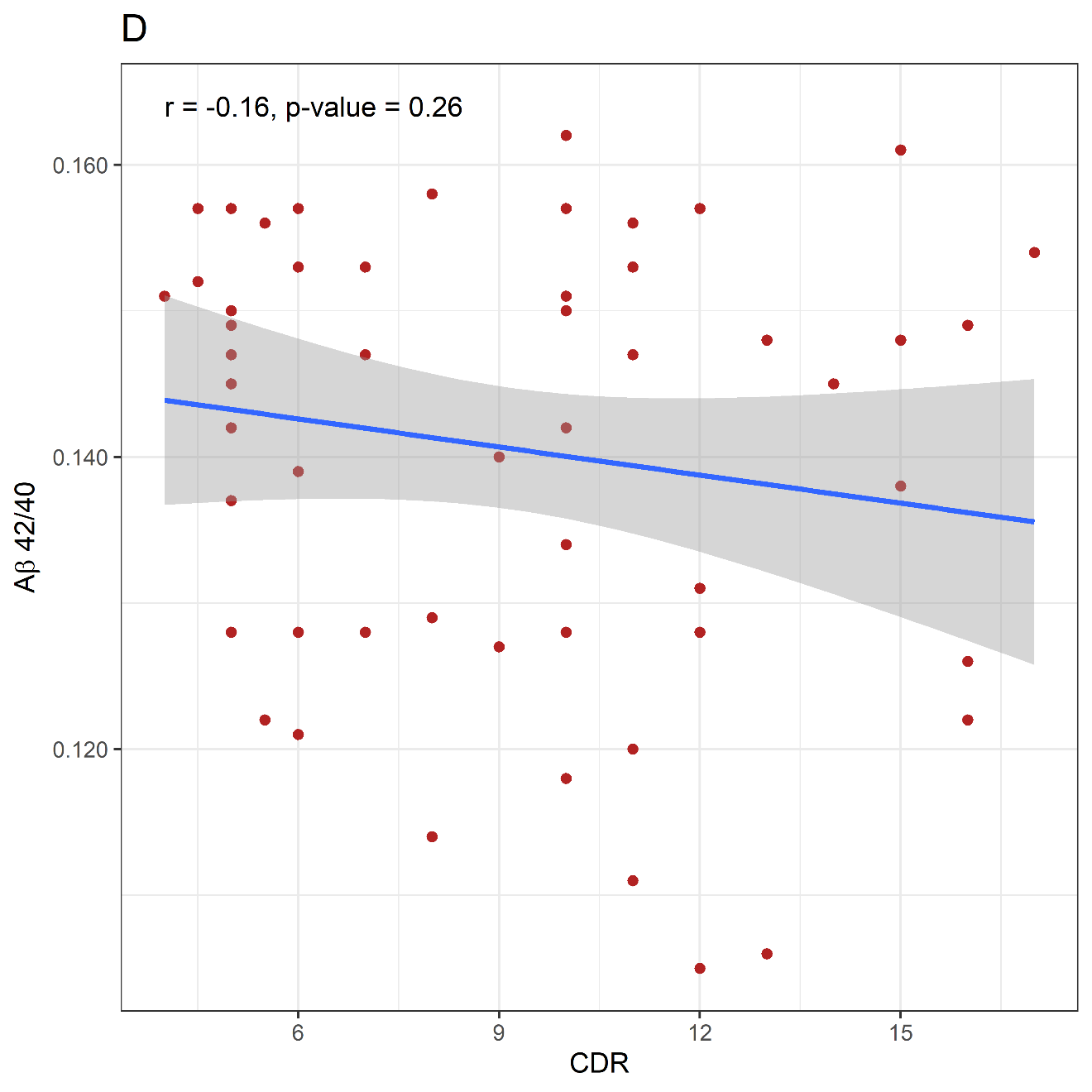


**References**

1. Weber DM, Kim JC, Goldman SM, Clarke NJ, Racke MK. New plasma LC-MS/MS assays for the quantitation of beta-amyloid peptides and identification of apolipoprotein E proteoforms for Alzheimer’s disease risk assessment. medRxiv. 2023:2023.11.20.23298532.

2. West T, Kirmess KM, Meyer MR, Holubasch MS, Knapik SS, Hu Y, et al. A blood-based diagnostic test incorporating plasma Aβ42/40 ratio, ApoE proteotype, and age accurately identifies brain amyloid status: findings from a multi cohort validity analysis. Mol Neurodegener. 2021;16(1):30.

3. Hu Y, Kirmess KM, Meyer MR, Rabinovici GD, Gatsonis C, Siegel BA, et al. Assessment of a plasma amyloid probability score to estimate amyloid positron emission tomography findings among adults with cognitive impairment. JAMA Netw Open. 2022;5(4):e228392.

4. Schindler SE, Bollinger JG, Ovod V, Mawuenyega KG, Li Y, Gordon BA, et al. High-precision plasma beta-amyloid 42/40 predicts current and future brain amyloidosis. Neurology. 2019;93(17):e1647-e59.

5. Schindler SE, Karikari TK, Ashton NJ, Henson RL, Yarasheski KE, West T, et al. Effect of Race on Prediction of Brain Amyloidosis by Plasma Abeta42/Abeta40, Phosphorylated Tau, and Neurofilament Light. Neurology. 2022;99(3):e245-e57.

6. Ovod V, Ramsey KN, Mawuenyega KG, Bollinger JG, Hicks T, Schneider T, et al. Amyloid beta concentrations and stable isotope labeling kinetics of human plasma specific to central nervous system amyloidosis. Alzheimers Dement. 2017;13(8):841-9.

7. Li Y, Schindler SE, Bollinger JG, Ovod V, Mawuenyega KG, Weiner MW, et al. Validation of Plasma Amyloid-beta 42/40 for Detecting Alzheimer Disease Amyloid Plaques. Neurology. 2021.

8. Janelidze S, Teunissen CE, Zetterberg H, Allue JA, Sarasa L, Eichenlaub U, et al. Head-to-Head Comparison of 8 Plasma Amyloid-beta 42/40 Assays in Alzheimer Disease. JAMA Neurol. 2021.

9. Rabe C, Bittner T, Jethwa A, Suridjan I, Manuilova E, Friesenhahn M, et al. Clinical performance and robustness evaluation of plasma amyloid-beta(42/40) prescreening. Alzheimers Dement. 2023;19(4):1393-402.

10. Zicha S, Bateman RJ, Shaw LM, Zetterberg H, Bannon AW, Horton WA, et al. Comparative analytical performance of multiple plasma Abeta42 and Abeta40 assays and their ability to predict positron emission tomography amyloid positivity. Alzheimers Dement. 2022.

11. Kirmess KM, Meyer MR, Holubasch MS, Knapik SS, Hu Y, Jackson EN, et al. The PrecivityAD test: Accurate and reliable LC-MS/MS assays for quantifying plasma amyloid beta 40 and 42 and apolipoprotein E proteotype for the assessment of brain amyloidosis. Clin Chim Acta. 2021;519:267-75.

12. Pascual-Lucas M, Allue JA, Sarasa L, Fandos N, Castillo S, Terencio J, et al. Clinical performance of an antibody-free assay for plasma Abeta42/Abeta40 to detect early alterations of Alzheimer's disease in individuals with subjective cognitive decline. Alzheimers Res Ther. 2023;15(1):2.

13. Janelidze S, Palmqvist S, Leuzy A, Stomrud E, Verberk IMW, Zetterberg H, et al. Detecting amyloid positivity in early Alzheimer's disease using combinations of plasma Abeta42/Abeta40 and p-tau. Alzheimers Dement. 2022;18(2):283-93.

14. Nakamura A, Kaneko N, Villemagne VL, Kato T, Doecke J, Dore V, et al. High performance plasma amyloid-beta biomarkers for Alzheimer's disease. Nature. 2018;554(7691):249-54.

15. Keshavan A, Pannee J, Karikari TK, Rodriguez JL, Ashton NJ, Nicholas JM, et al. Population-based blood screening for preclinical Alzheimer's disease in a British birth cohort at age 70. Brain. 2021;144(2):434-49.

16. Brand AL, Lawler PE, Bollinger JG, Li Y, Schindler SE, Li M, et al. The performance of plasma amyloid beta measurements in identifying amyloid plaques in Alzheimer's disease: a literature review. Alzheimers Res Ther. 2022;14(1):195.

17. Bun S, Ito D, Tezuka T, Kubota M, Ueda R, Takahata K, et al. Performance of plasma Abeta42/40, measured using a fully automated immunoassay, across a broad patient population in identifying amyloid status. Alzheimers Res Ther. 2023;15(1):149.
